# Effectiveness of Rehabilitation for Osteoarthritis of the Knee: A Scoping Review of Network Meta-Analyses

**DOI:** 10.7759/cureus.57661

**Published:** 2024-04-05

**Authors:** Takashi Kitagawa, Takumi Denda, Wataru Okuyama, Ryo Miyachi, Keisuke Nakamura

**Affiliations:** 1 Department of Physical Therapy, School of Health Sciences, Shinshu University, Matsumoto, JPN; 2 Department of Rehabilitation, Tsukada Orthopedics, Tsuchiura, JPN; 3 Department of Physical Therapy, Faculty of Health and Medical Sciences, Hokuriku University, Kanazawa, JPN

**Keywords:** conservative treatment, quality of life, physical functional performance, pain, electric stimulation therapy, exercise therapy

## Abstract

Background: Although an increasing number of network meta-analyses have been conducted on the effectiveness of conservative therapy for knee osteoarthritis, these may have been poorly planned and executed.We aimed to review the qualities of a comprehensive set of network meta-analyses on rehabilitation therapies for knee osteoarthritis and provide an overview of the effectiveness of each therapy.

Methods: The eligibility criteria were as follows: (i) conservative rehabilitation was the primary treatment in the intervention group, (ii) included patients were diagnosed with knee osteoarthritis, and (iii) patient groups were aged ≤75 years, and rehabilitation interventions comprised exercise, orthotic, or physical therapies or patient education. Two independent reviewers screened the titles and abstracts of the identified records and selected the eligible reviews; their full texts were further assessed for eligibility. Then, a checklist derived from the Preferred Reporting Items for Systematic Reviews and Meta-Analyses (PRISMA) extension statement for the reporting of systematic reviews incorporating network meta-analyses of healthcare Interventions was used to validate the completeness of the reporting of each network meta-analysis. Furthermore, the statistical and outcome data regarding the quality of life, knee joint function and pain, adverse events, and physical functions were extracted using a customized spreadsheet.

Results: Overall, 2701 titles and abstracts were screened, and eight network meta-analyses were ultimately selected. Nearly all reviews adequately addressed the PRISMA extension checklist, and the completeness of reporting was adequate; therefore, all expected information could be extracted. However, the methodology used to confirm the transitivity assumption was insufficient in many reviews. The following interventions were effective in reducing pain and improving physical function: (i) strengthening, flexibility, aerobic, and mind-body exercises, (ii) pulsed ultrasound, (iii) focused and radial extracorporeal shockwave therapy, and (iv) continuous ultrasound. The following interventions were effective in improving the quality of life: (i) strengthening, (ii) mixed, and (iii) mind-body exercises.

Conclusions: Our results suggested that exercise therapies, including muscle-strengthening, aerobic, flexibility, and mind-body exercises, are likely to be effective for pain relief and functional improvement in knee osteoarthritis. This may be the first review to provide a comprehensive perspective for considering priorities for future rehabilitation interventions for knee osteoarthritis.

## Introduction and background

Globally, osteoarthritis is the most common degenerative joint disease that causes disability in daily living activities [1]. Knee osteoarthritis (KOA) is one of the most common causes of chronic pain and disability worldwide [2]. Conservative therapy is one of the most commonly used treatments for KOA [2,3]. Accordingly, the number of systematic reviews of randomized controlled trials (RCTs) for conservative therapy for KOA has increased in recent years [4-6]. Although these reviews have identified some adverse events, such as arthralgia or skin/subcutaneous tissue disorders associated with conservative therapy, the benefits of this form of therapy remain evident [7]. Conservative therapy includes a wide range of treatment factors (e.g., exercise therapy, pharmacotherapy, patient education, and self-management programs) [1,5] that are recommended in the clinical practice guidelines by the Royal Dutch Society for Physical Therapy in the Netherlands and by a panel of experts in Ottawa, Canada [8-11].

In addition to the existing systematic reviews, network meta-analyses (NMAs) have been conducted to integrate the effectiveness of treatment interventions; unlike traditional pairwise comparisons, these NMAs have enabled an examination of the effects of multiple treatment interventions [12]. NMAs enable the estimation of effects not only directly through comparisons among the treatments but also indirectly through statistical methods [13]. For example, in a typical clinical trial (such as an RCT), two-armed or three-armed groups can be exclusively compared. In contrast, NMAs allow for the estimation of both the degree of effectiveness of each conservative treatment for KOA and the priority ranking of these treatments [14].

Reports of NMAs on KOA, particularly those on comparisons of pharmacotherapy interventions, have increased in recent years [15-18]. Furthermore, NMAs evaluating conservative therapy for KOA have also increased slightly [19,20]; however, these NMAs may have been poorly planned and executed [21].

Reporting guidelines and Cochrane methodology are good tools of methodological rigor; however, their usage was not always observed in the existing NMAs [22,23]. An overview of published NMAs and an assessment of their methodological features may help guide future research. Thus, this scoping review aimed to assess and summarize the qualities of a comprehensive set of NMAs on the effectiveness of rehabilitation interventions for patients with KOA and provide an overview of the effectiveness of each conservative therapy.

## Review

Methods

Study Protocol and Eligibility Criteria

We developed a priori protocol for this review, which was prospectively registered with the Open Science Framework on March 2, 2022 (https://osf.io/zv2ps/). NMAs that examined the effects of rehabilitation interventions in RCTs were included if they met the following criteria: (i) conservative rehabilitation was the primary treatment in the intervention group, (ii) the included patients were diagnosed with KOA (Kellgren and Lawrence grades I-III, Ⅰ: doubtful narrowing of the joint space with possible osteophyte formation, Ⅱ: possible narrowing of the joint space with definite osteophyte formation, Ⅲ: definite narrowing of joint space, moderate osteophyte formation, some sclerosis, and possible deformity of bony ends) by a physician based on imaging or clinical findings, (iii) the analyzed patient groups had a mean (or median) age of ≤75 years, and (iv) the rehabilitation interventions comprised exercise therapy (muscle-strengthening training and aerobic exercises), orthotic therapies (such as use of insoles), various physical therapies (such as electrotherapies), or patient education [2,8]. We excluded the following: (i) NMAs of studies involving patients scheduled for or undergoing surgical treatment, (ii) NMAs of interventional studies on cases of acute inflammation, (iii) NMAs of individual participant data, and (iv) NMAs of non-RCTs. The control group received usual care (exercise instructions only, advice to maintain daily activity level only, light aerobic exercise of <10 minutes, light stretching not aimed at improving flexibility, or light-impact strength training not aimed at achieving muscle hypertrophy/strengthening), placebo treatment (e.g., sham intervention of electrophysiotherapy), or no intervention.

The primary outcomes were quality of life (measured using the 12-Item Short Form Survey (SF-12) or the 36-Item Short-Form Survey (SF-36)), knee joint function (measured using the Western Ontario and McMaster Universities Osteoarthritis Index (WOMAC) or the Knee Injury and Osteoarthritis Outcome Score (KOOS)), and adverse events (such as exacerbation of pain or falls). The secondary outcomes were pain (measured on the numerical rating scale (NRS) or visual analog scale (VAS)) and physical function (measured mostly in terms of gait speed, among others). No restrictions were imposed based on the region, race, or language.

Information Sources and Search Methods

To identify exercise trials, the following databases were searched on April 6, 2022: PubMed, Embase (via ProQuest Dialog), Cumulative Index to Nursing and Allied Health Literature, Web of Science, and Cochrane Central Register of Controlled Trials (via Cochrane Library). Additionally, we searched the OpenGrey platform to assess grey literature, which comprises literature produced outside of traditional academic publishing and distribution channels. Finally, the citation lists of the included articles were screened as part of “citation searching.” Full details of the search strategy are provided in the Open Science Framework.

Study Selection

Rayyan was used to remove duplicates and screen the articles [24]. Two reviewers (WO and TD) independently screened the titles and abstracts of the initially identified articles as well as the full texts of the subsequently eligible articles. Disagreements were resolved through consensus between the authors or through discussions with a third author (TK).

Data Charting Process and Data Items

Two authors (TK and TD) independently extracted data using a customized spreadsheet. The following information was collected from the included articles: title, authors, year of publication, nationality of the first author, number of studies and participants included in the NMA, patient inclusion and exclusion criteria, age range of the participants, treatment details in the intervention and control groups, outcomes of interventions, adverse event reports, and main findings of each review. The following NMA-related details were collected: the number of nodes, NMA framework, NMA software, and transitivity assessment strategy. Inconsistencies were resolved through consensus between TK and TD.

Critical Appraisal of Individual Sources of Evidence

A checklist derived from the Preferred Reporting Items for Systematic Reviews and Meta-Analyses (PRISMA) extension statement for the reporting of systematic reviews incorporating network meta-analyses of healthcare interventions (PRISMA-NMA) was used to validate the completeness of reporting for each NMA [22]. Two reviewers (TK and WO) independently assessed the NMAs to evaluate their methodological quality; disagreements were resolved through a consensus between the two.

Data Analysis and Presentation

The presented data comprise the general characteristics of the included studies. The methodological characteristics were summarized by two independent reviewers (TK and TD). Inconsistencies were resolved through discussions. A word cloud was generated to visualize the review terms, and a radar plot was used to highlight the relevant NMA reporting items.

Results

Description of the Included Studies and Patient Characteristics

**Figure 1:**
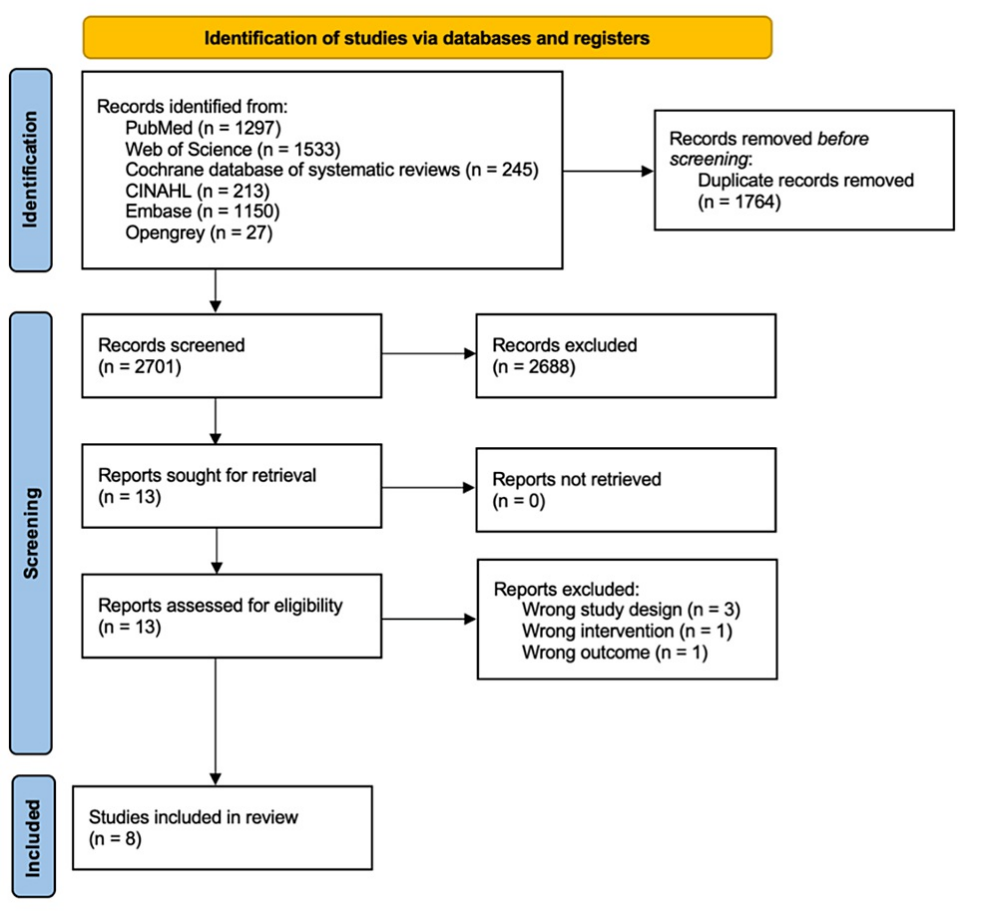
Flowchart of the search strategy and results. CINAHL, Cumulative Index to Nursing & Allied Health

The database search identified 4465 records. After the removal of duplicates, the titles and abstracts of the remaining 2701 records were screened; among these, 2688 did not meet the eligibility criteria. Subsequently, the full texts of the remaining 13 articles were screened for eligibility. Three NMAs were excluded because the study designs did not meet our eligibility criteria, one was excluded because the interventions did not meet the same, and another one was excluded because the outcomes considered did not meet the eligibility criteria; therefore, eight studies were finally selected for the review (Figure 1) [19,20,25-30].

The characteristics of the included NMAs are presented in Table 1. These were published in the following years: 2013 (n=1) [25], 2014 (n=1) [26], 2015 (n=1) [27], 2019 (n=2) [19,20], 2021 (n=1) [28], and 2022 (n=2) [29,30]. Furthermore, these NMAs were conducted in Australia [28], China [26,27], Georgia [19], Norway [30], Taiwan [29], and the United Kingdom [20,25]. In four NMAs, the participants exclusively had KOA [26-29]; in the remaining four NMAs, the participants presented with KOA and osteoarthritis of other joints [19,20,25,30].

**Table 1:** Characteristics of the participants in each included review (N=8) † The number of studies and sample sizes varied by the outcome. ‡ Patients with self-reported knee osteoarthritis, determined on the basis of chronic knee joint pain with or without radiographic confirmation, were included.

Authors (year)	Authors’ country	Number of studies and patients	Inclusion criteria for the patients	Age range (years)
Zhang et al. (2019) [19]	Georgia	32 studies (n=3228)	Patients aged ≥60 years with osteoarthritis; no restrictions based on sex or the affected joint	65–83
Goh et al. (2019) [20]	United Kingdom	30–78 studies (n=2073–5733)^†^	Patients with knee osteoarthritis, hip osteoarthritis, or a combination of knee and hip osteoarthritis (as determined by clinical or radiographic examination)	41–84
Uthman et al. (2013) [25]	United Kingdom	60 studies (n=8037)	Patients with an established clinical or radiographic diagnosis of knee or hip osteoarthritis	Not reported
Zeng et al. (2014) [26]	China	8 studies (n=525)	Patients with knee osteoarthritis	53.6–69.4
Zeng et al. (2015) [27]	China	20 studies (n=995)	Patients with knee osteoarthritis	45–85.9
Hall et al. (2021) [28]	Australia	13 studies (n=897)	Patients with knee osteoarthritis with clinical changes (history and physical examination); clinical and laboratory changes; and clinical, laboratory, and radiographic changes^‡^	56.1–71.9
Liao et al. (2022) [29]	Taiwan	70 studies (n=5980)	Patients with a symptomatic or radiographic diagnosis of knee osteoarthritis	40.1–80.3
Smedslund et al. (2022) [30]	Norway	7 studies (sample size details were not provided)	Patients with knee, hip, or hand osteoarthritis	Not reported

**Figure 2:**
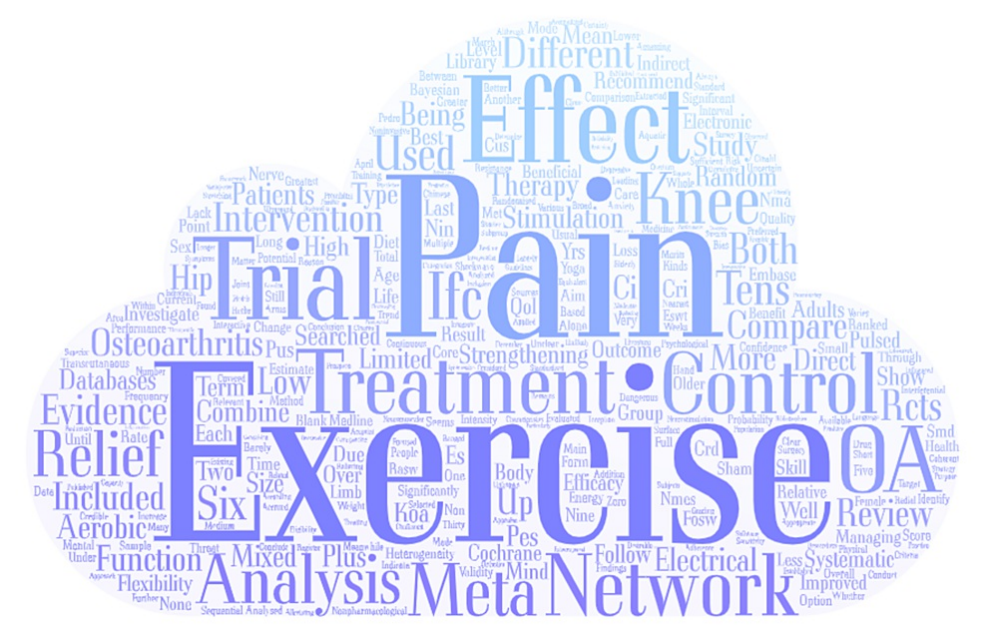
Word cloud generated from the included study words

Table 2 displays the treatment characteristics and participant outcomes for all eight NMAs. Five NMAs compared exercise therapies [19,20,25,28,30], while three compared various physical agents [26,27,29]. Quality of life was measured using SF-12, RAND 36, or SF-36 [20,28]. The WOMAC physical function subscale [20,26,27,29], KOOS activities of daily living subscale [20,25,29], and Lequesne index [26,29] were used to assess the outcomes related to knee function. A few adverse events were identified in three NMAs; these included skin rash development, blood pressure spikes, and an electric shock/stinging sensation. All adverse events were attributed to interventions with electrophysical modalities. In six NMAs, the VAS [19,20,25-27,29] was used to assess pain-related outcomes; additionally, the NRS [19,27], WOMAC pain subscale [19,20,26,27], KOOS pain subscale [19,27], and SF-36 pain subscale [19,20] were used to assess pain. The most common outcome related to physical function was walking speed [20,26]. The word cloud generated from the titles and abstracts of the eight NMAs is displayed in Figure 2; “pain” and “exercise” were the common words in the included NMAs.

**Table 2:** Treatments and outcomes in the included review articles (N=8) † As a replacement for the WOMAC function scores when a study did not consider them, the WOMAC total scores, Lequesne Index, or other scores from functional measurement scales were applied to the analysis. ESWT, extracorporeal shock wave therapy; VAS, visual analog scale (the score ranges from 0 to 100, 0 means no pain and 10 means worst pain); WOMAC, Western Ontario and McMaster Universities Osteoarthritis Index (the scores for each subscale are summed, with a possible score range of 0 to 20 for pain, 0 to 8 for stiffness, and 0 to 68 for physical function, with higher scores indicating worse pain, stiffness, and functional limitations); SF-36, short-form 36; SF-12, short-form 12 (SF-36 or SF-12 scores range from 0 to 100, with higher scores indicating better functioning in the physical and mental health domains); KOOS, Knee Injury and Osteoarthritis Outcome Score (the score ranges from 0 to 210, 0 is the best score indicating the absence of disability)

Authors (year)	Treatments in the intervention group	Treatments in the control group	Outcomes considered for evaluating the effect of interventions
Zhang et al. (2019) [19]	Nonpharmacological interventions for pain relief	Not mentioned	Pain intensity
Goh et al. (2019) [20]	Muscle strengthening, aerobic, or flexibility/neuro-motor skills training	Routine medical care or no specific interventions	The primary outcome was pain, whereas the secondary outcomes were self-reported function, objective performance, and quality of life.
Uthman et al. (2013) [25]	Exercise interventions (on land or in water)	Exercise with other methods or no exercise	Scores from at least one assessment of self-reported pain and function must be included in the study.
Zeng et al. (2014) [26]	Pulsed ultrasound or continuous ultrasound	Blank or sham	As a measure of function, the WOMAC function subscale was preferred.^†^
Zeng et al. (2015) [27]	Interferential current stimulation, neuromuscular electrical stimulation, non-invasive interactive neurostimulation, pulsed electrical stimulation, or transcutaneous electrical nerve stimulation	Blank or sham	Examples of pain scores include the VAS, WOMAC, and present pain intensity scores.
Hall et al. (2021) [28]	Strengthening, aerobic exercises, mind-body exercises, or mixed	Described based on the authors’ descriptions.	SF-36 (mental health summary) and SF-12 (mental health summary) scores
Liao et al. (2022) [29]	ESWT alone or in combination with other noninjective treatments	Placebo treatment, an ESWT of a relatively low dosage, or a non-ESWT intervention	Knee function (e.g., WOMAC score, KOOS, Lequesne index, and Lysholm Knee Scoring Scale score) and pain (e.g., VAS score)
Smedslund et al. (2022) [30]	Exercises, mind-body exercises, passive treatment, and orthotics	Standard treatment, placebo treatment, no interventions, or other interventions	Pain, physical function, patient’s global assessment of disease activity, and quality of life

Completeness of Reporting

**Figure 3:**
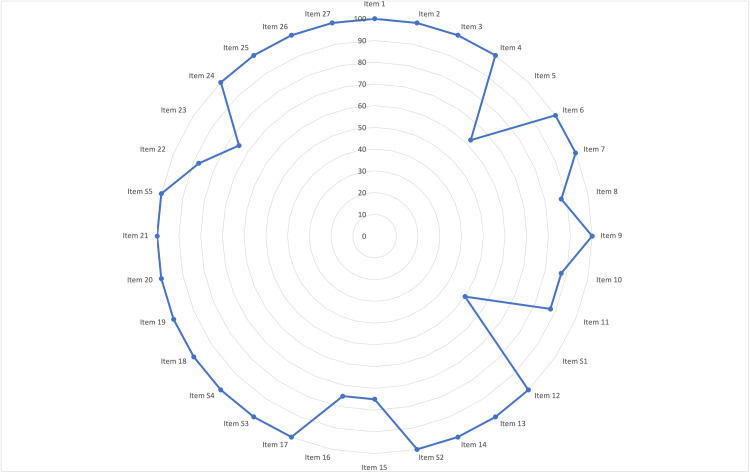
Reporting items relevant to network meta-analyses

The percentages of the included NMAs that adequately addressed each of the 32 items on the PRISMA-NMA checklist are shown in Figure 3. Detailed descriptions of the assessments used in this review are provided in Table 3. Although three of the eight included NMAs were published before the publication of the PRISMA-NMA checklist, the overall completeness of the reports was generally high. The protocols and registration (checklist item 5; 62.5%) were mentioned for five of the eight targeted NMAs; conversely, the method for examining the shape of the networks was described in only four of the eight targeted NMAs (checklist item S1; 50%). Additionally, reporting on the assessment of the overall risk of bias (checklist item 15), methodology used for additional analyses (checklist item 16), and results of the additional analyses (checklist item 23) was inadequate (75% for each of these).

**Table 3:** PRISMA-NMA assessments for each included review PRISMA-NMA, Preferred Reporting Items for Systematic Reviews and Meta-analyses Extension Statement for Reporting of Systematic Reviews Incorporating Network Meta-analyses of Health Care Interventions; Y, yes

Authors (year)	Reporting of the assessment for introduction and methods																															
	1	2	3	4	5	6	7	8	9	10	11	S1	12	13	14	S2	15	16	17	S3	S4	18	19	20	21	S5	22	23	24	25	26	27
Zhang et al. (2019) [19]	Y	Y	Y	Y	Y	Y	Y	Y	Y	Y	Y	Y	Y	Y	Y	Y	Y	Y	Y	Y	Y	Y	Y	Y	Y	Y	Y	Y	Y	Y	Y	Y
Goh et al. (2019) [20]	Y	Y	Y	Y	Y	Y	Y	Y	Y	Y	Y		Y	Y	Y	Y		Y	Y	Y	Y	Y	Y	Y	Y	Y	Y	Y	Y	Y	Y	Y
Uthman et al. (2013) [25]	Y	Y	Y	Y	Y	Y	Y	Y	Y	Y	Y		Y	Y	Y	Y		Y	Y	Y	Y	Y	Y	Y	Y	Y			Y	Y	Y	Y
Zeng et al. (2014) [26]	Y	Y	Y	Y		Y	Y	Y	Y	Y			Y	Y	Y	Y	Y		Y	Y	Y	Y	Y	Y	Y	Y	Y	Y	Y	Y	Y	Y
Zeng et al. (2015) [27]	Y	Y	Y	Y		Y	Y	Y	Y	Y	Y		Y	Y	Y	Y	Y	Y	Y	Y	Y	Y	Y	Y	Y	Y	Y	Y	Y	Y	Y	Y
Hall et al. (2021) [28]	Y	Y	Y	Y		Y	Y	Y	Y	Y	Y	Y	Y	Y	Y	Y	Y		Y	Y	Y	Y	Y	Y	Y	Y	Y		Y	Y	Y	Y
Liao et al. (2022) [29]	Y	Y	Y	Y	Y	Y	Y	Y	Y	Y	Y	Y	Y	Y	Y	Y	Y	Y	Y	Y	Y	Y	Y	Y	Y	Y	Y	Y	Y	Y	Y	Y
Smedslund et al. (2022) [30]	Y	Y	Y	Y	Y	Y	Y		Y		Y	Y	Y	Y	Y	Y	Y	Y	Y	Y	Y	Y	Y	Y	Y	Y	Y	Y	Y	Y	Y	Y

Methodological Reporting

The methodological characteristics of the included studies are listed in Table 4. The network consisted of 3-22 nodes (median, 9.5). Bayesian models were used for the NMA analyses in four NMAs (50%) [20,25-27], whereas a frequentist approach was used in the remaining four NMAs (50%) [25,28-30]. Meanwhile, meta-regression was performed in three NMAs. For NMA, Stata (StataCorp LLC, College Station, Texas, United States), R (R Foundation for Statistical Computing, Vienna, Austria), and WinBUGS were used in five [19,20,26-28], four [26,27,29,30], and four [20,25-27] reviews, respectively. The transitivity assessment strategy was clearly explained in two NMAs [29,30].

**Table 4:** Methodological characteristics of the included reviews (n=8) † The Markov Chains Monte Carlo method was used to obtain the pooled effect sizes. NMA, network meta-analysis; BUGS, Bayesian inference Using Gibbs Sampling

Authors (year)	Nodes in the network	Framework for NMA	Statistical model for NMA	Software for NMA	Strategy for assessing transitivity
Zhang et al. (2019) [19]	12	Not mentioned	Not mentioned	Stata	Inappropriate methods
Goh et al. (2019) [20]	6	Frequentist and Bayesian	Meta-regression	WinBUGS and Stata	Inappropriate methods
Uthman et al. (2013) [25]	13	Bayesian	Meta-regression	WinBUGS	Inappropriate methods
Zeng et al. (2014) [26]	3	Bayesian	Not mentioned	WinBUGS, R, and Stata	Inappropriate methods
Zeng et al. (2015) [27]	7	Bayesian	Other^†^	WinBUGS, R, and Stata	Inappropriate methods
Hall et al. (2021) [28]	6	Frequentist	Not mentioned	Stata	Inappropriate methods
Liao et al. (2022) [29]	22	Frequentist	Meta-regression	R	Comparing the distribution of potential effect modifiers across comparisons
Smedslund et al. (2022) [30]	17	Frequentist	Not mentioned	R	Comparing the distribution of potential effect modifiers across comparisons

Discussion

Summary of Results

In this scoping review, we assessed and summarized the methodological qualities of NMAs that examined the effectiveness of rehabilitation interventions for KOA. The majority of the eight included reviews were performed using appropriate methodology. However, inadequate reporting was observed for several items (particularly protocol registration).

Characteristics of the Included Reviews

Most of the articles included in this scoping review were published in the past decade. Some NMAs included a mixture of patients with hip and wrist osteoarthritis; however, five reviews exclusively included patients with KOA. The ages of all patients ranged between 40 and 85 years but did not appear to differ significantly from the ages of participants in other clinical studies on KOA. The following interventions were effective in reducing pain: strength exercises [19,20,25], flexibility exercises [20,25], aerobic exercises [25,30], mind-body exercises [20], pulsed ultrasound (PUS) [26], continuous ultrasound (CUS) [26], and extracorporeal shockwave therapy (ESWT) [29]. Exercise therapy is widely recognized as an effective means of treating KOA pain because increased lower limb strength may reduce internal knee forces and pain [31]. Therefore, this perspective is unlikely to change significantly. Additionally, ultrasound therapy is expected to reduce pain thresholds in vivo through thermal and non-thermal effects [32]. However, negative findings were obtained regarding the effectiveness of electrophysical modalities [8]. Several individual RCTs have revealed significant differences in the effectiveness between electrophysical modalities and controls; however, these studies are of insufficient quality or have small effect sizes. Moreover, a recently published large RCT found that transcutaneous electrical nerve stimulation (TENS) did not affect knee joint pain or function [33]; therefore, there is little need to examine the effects of TENS using meta-analyses or other methods, and exercise therapy should be the first choice of treatment for pain reduction.

The following interventions were suggested to be effective for improving physical function: mind-body exercises [20], combined intervention with strengthening, flexibility, and aerobic exercises [20], PUS [26], and ESWT [29]. Exercise therapy is known to be effective for patients with KOA in the conservative phase of treatment; however, the quality of evidence on the effectiveness of exercise therapy for patients with KOA before total knee arthroplasty is low. Nevertheless, exercise therapy does have a moderate effect on the physical function. Therefore, the extent to which exercise therapy is indicated for patients with severe osteoarthritis requires individualized consideration [8]. Regarding the type of ultrasound therapy, the finding that PUS is more effective than CUS is supported by findings from previous RCTs as well [34]. As a possible mechanism, PUS induces the proliferation of chondrocytes and matrix production in human articular cartilage [26]. The benefits of including PUS in conventional care have been demonstrated by previous studies; however, the effect sizes were very small and did not exceed the minimal clinically significant difference [35]. Therefore, clinical applications must consider individual factors, such as cost-effectiveness and patient preferences. While the effectiveness of various types of exercise therapy has been consistently demonstrated, the capacity-response relationship between exercise therapy and pain and physical function remains unclear [36]. Thus, further detailed examinations of the appropriate intensity and frequency of exercise therapy are needed.

The following interventions were shown to be effective in improving the quality of life: strengthening, mixed, and mind-body exercises [28]. According to recent guidelines [9], the recommended strengthening exercise programs include the following: (i) home-based progressive strengthening exercise programs, (ii) group-based supervised progressive strengthening and coordination exercise programs, (iii) progressive resistance exercise programs for the knees and hip muscles, (iv) lower extremity strengthening exercise programs, (v) concentric-eccentric isokinetic and isometric exercise programs, and (vi) strengthening exercise programs that include patient education. Specific examples of aerobic exercise programs include individual- and group-supervised aerobic and strengthening exercise programs and cycling exercise programs [11]. Tai Chi Qigong exercise programs were recommended for mind-body exercises, whereas Hatha Yoga and sun-style Tai Chi exercise programs were considered insufficiently effective and were not recommended [9]. Considering the individuality of a patient’s physical function, lifestyle, and goals, it is advisable to provide them with information on these programs and conduct shared decision-making [37].

Completeness of Reporting of the Included Reviews

Protocol and registration: A previous review revealed that only 13% of the 494 scoping reviews analyzed reported a predetermined protocol [38]. Another review revealed that 41.6% of 89 NMAs of complementary and alternative medicine interventions mentioned the protocol and registration details [12]. The reporting rate for the NMAs included in the present review was relatively good (62.5%); this was unsurprising given that papers with insufficient information on protocol and registration were mostly published between 2014 and 2015, i.e., before the PRISMA-NMA checklist was published. The subsequent trend of NMAs providing adequate information suggested that articles on NMAs published since 2015 (i.e., when the PRISMA-NMA checklist [22] was published) complied with the checklist. It is important to register new NMA protocols, once written, to prevent selective outcome reporting bias.

Review of network geometry: Despite the network structures presented in all eight reviews, approximately half of these reviews lacked methodological explanations of the network geometries. It is important to understand the pattern of differences among different interventions when randomized trials are conducted under the same conditions. To avoid comparator preference bias, it would have been desirable for the authors of these reviews to state in the methods section that sufficient consideration was given to each network [39].

Risk of bias across studies: Two reviews provided insufficient or no explanation of the risk of bias across studies in their methods sections [20,25]. Furthermore, one of these reviews mentioned publication bias in the results section [25]. The identification of publication bias is reported to be more complex in NMAs [22], although the use of comparison-adjusted funnel plots [40] or regression methods and selection models [41] is encouraged. Alternatively, a contour-enhanced funnel plot can be used [42].

Additional analyses: No methodological descriptions of additional analyses or their results were provided in two reviews [26,28]. When enough studies are included, additional analyses may provide helpful information in addition to the primary analysis. Specific methods, such as meta-regression analyses to adjust for covariates [43], subgroup analyses, and sensitivity analyses, should be considered to confirm the robustness of the integrated results [23]. With the release of PRISMA-NMA in 2015 [22], the completeness of reports on NMAs is expected to increase in the future with increased visibility.

Methodological Characteristics of the Included Studies

Number of nodes: The median number of nodes in the NMAs analyzed in this study was 9.5; this is generally consistent with the median number of 8 reported in a recent analysis of several NMA papers [44]. This node number is considered appropriate because analyses with too many nodes are less visible and may complicate the interpretation of the results.

Frameworks of the analysis: In the included reviews, the Bayesian (n=4 [50%]) and frequentist (n=4 [50%]) frameworks were used for analyses. However, previous reviews of a larger number of NMAs revealed that the Bayesian framework seemed more popular than the frequentist framework (usage: 66.7% vs. 32.2% in one study [12] and 51% vs. 20% in another study [21]).

Statistical analyses: Meta-regression was used for statistical analysis in three reviews [20,25,29]. As with standard meta-regression, if the number of trials is relatively smaller than the number of treatment comparators, the analysis will be characterized by low power [45]. However, if the number of included studies is sufficient, a meta-regression analysis can examine the effect modifiers for each trial's treatment effect [46].

Software for statistical analysis: The NMAs included in this review were analyzed using WinBUGS, R, and Stata; however, OpenBUGS was a popular choice as well [21]. Practitioners need to access these four software and work closely with biostatisticians [23].

Method for evaluating transitivity: Two of the eight NMAs included in this review confirmed the transitivity assumption by assessing potential effect modifiers [29,30]. In a recent review of 45 Cochrane NMA protocols [21], transitivity assumptions were considered in the reporting of the inclusion criteria in approximately half of the cases; furthermore, possible effect modifiers were specified in 78% of the cases. While planning future NMAs, a more careful evaluation of the effect modifiers and transitivity assumptions should be considered [47].

Study Limitations

This study has several limitations. First, the interventions in each NMA varied considerably. The eight NMAs included five exercise therapies and three electrophysical modalities; however, there was a great deal of diversity within each concept as well. This review set the definition of intervention to the broad concept of rehabilitation, although manual and orthotic therapies were omitted. Second, two of the three NMAs on electrophysical modalities summarized evidence that was approximately 10 years old. Appropriate reconfiguration of similar populations, intervention, control, and outcomes (PICOs) and implementation of NMAs may yield different results and new information. Third, this review included five NMAs for exercise therapy, all of which had a variety of PICOs. If the effectiveness of exercise therapy is more adequately examined in the future, the intervention should be specific, the participants should be localized and stratified, and the outcomes should be segmented. Finally, it should be noted that this is a review of NMAs, and data extraction of individual RCTs and analyses based on those data were not available. Because of the potential risk of over-interpretation due to the possible overlap of individual RCTs within each NMA, future studies should be based on individual data extraction and analysis.

## Conclusions

Multiple previous NMAs have revealed the relative priority ranking of effectiveness for each rehabilitation intervention for KOA. Exercise therapies, such as muscle-strengthening, aerobic, flexibility, and mind-body exercises, are likely to be effective in reducing pain, improving physical function, and enhancing the quality of life of patients with KOA. This review may serve as the first paper to provide a comprehensive perspective when considering priorities for future rehabilitation interventions for KOA.
